# Injectable Dendritic Hydrogels Curable by High-Energy Visible Light for Cell Delivery in Bone Regeneration

**DOI:** 10.1021/acs.chemmater.5c00063

**Published:** 2025-04-16

**Authors:** Noemi Molina, Francesco Torelli, Samih Mohamed-Ahmed, Daniel J. Hutchinson, Cecilie Gjerde, Ahmad Rashad, Kamal Mustafa, Michael Malkoch

**Affiliations:** 1 Department of Fibre and Polymer Technology, KTH Royal Institute of Technology, Teknikringen 56-68, 100 44 Stockholm, Sweden; 2 Department of Clinical Dentistry, University of Bergen, Årstadveien 19, 5009 Bergen, Norway

## Abstract

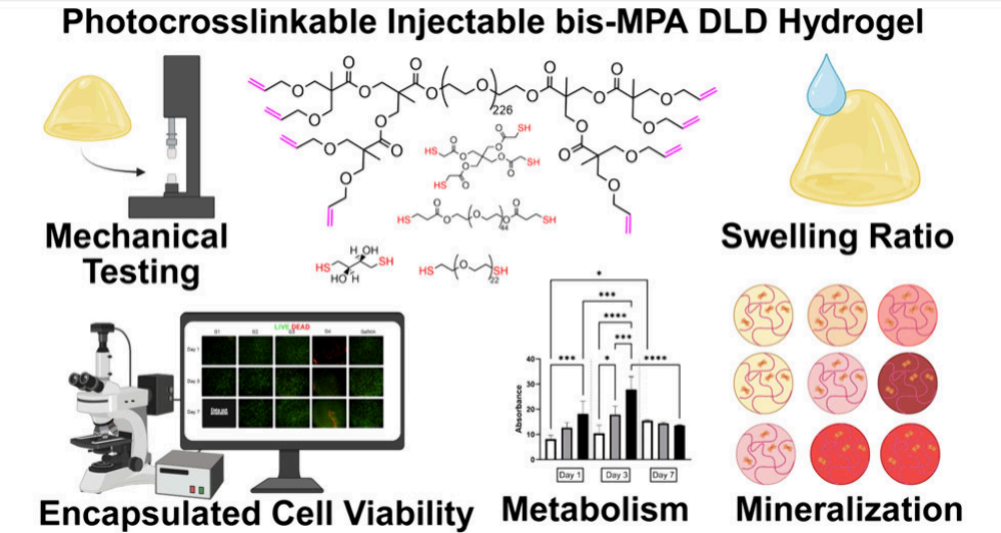

Hydrogels loaded with bone marrow mesenchymal stem cells (BMSCs) have emerged as a promising alternative to grafting for bone regeneration in critical-sized fractures and defects. Here, we present a platform for an injectable bone scaffold hydrogel that cures in situ via high-energy visible (HEV) light-induced thiol–ene coupling (TEC) chemistry. The hydrogel platform consists of branched allyl-functionalized dendritic–linear–dendritic (DLD) copolymers, constructed from poly(ethylene glycol) (PEG) and 2,2-bis(hydroxymethyl)propionic acid (bis-MPA), and thiolated cross-linkers. The hydrogels’ stability, swelling behavior, and modulus can be finely tuned by varying the DLD generation, cross-linker valency and length, and dry weight content. In vitro cytocompatibility assessments reveal that the platform supports BMSC viability and interactions, comparable to those of a control hydrogel gelatin methacryloyl (GelMA). Further evaluation of the best-performing hydrogels composed of the second-generation PEG10k-G2-BAPA DLD with either dl-dithiothreitol (DTT) or PEG1k-SH cross-linkers demonstrates similar cell metabolic activity to GelMA after 7 days and significant calcium deposition after 14 and 21 days in osteogenic medium. The preferred gel, incorporating DTT, also shows a high capacity for functionalization with inorganic fillers (e.g., hydroxyapatite) and biopolymers (e.g., collagen). Collectively, the results highlight, for the first time, the broad potential of bis-MPA-based dendritic hydrogels as versatile soft biomaterials for regenerative medicine applications.

## Introduction

Strategies employing mesenchymal stem cells (MSCs) and scaffolding biomaterials for bone regeneration have recently gained attention as a means to overcome the limitations of bone grafting, the current gold standard for treating bone defects.^1,2^ MSCs are highly valued for their remarkable ability to differentiate into various cell lineages, including osteogenic cells, and their secretion of a diverse array of bioactive molecules such as growth factors, cytokines, and chemokines that enhance regeneration and healing processes.^3^ Among the various sources of MSCs, those derived from bone marrow (BMSCs) have demonstrated superior osteogenic potential compared to MSCs from other tissues such as adipose tissue.^4,5^ Consequently, BMSCs are now the most commonly used cells in bone regenerative applications utilizing tissue engineering strategies, in both preclinical and clinical settings.

Hydrogels have emerged as promising scaffolds for cell encapsulation in tissue engineering, largely due to their high-water content and tunable physicochemical properties, such as elasticity and degradability. These properties can be finely adjusted through the design of natural or synthetic polymers that constitute the hydrogel network.^6,7^ Natural hydrogels, derived from materials such as gelatin, alginate, and chitosan, offer excellent biocompatibility and biodegradability.^8,9^ Synthetic hydrogels provide greater control over mechanical properties and degradation rates.^9^ Degradability is crucial for allowing for the replacement of the scaffold by healthy tissue during the healing process,^10^ while the elasticity of the gel needs to be precisely tuned to support cell fate specification.^11^ Synthetic polymers commonly used in medical hydrogels include polycaprolactones, polylactic acid, poly(*N*-isopropylacrylamide), and poly(ethylene glycol) (PEG), the last being favored for its hydrophilicity and biocompatibility.^12,13^ A key feature for clinical applications is the incorporation of chemical groups that enable on-demand covalent cross-linking of hydrogel networks through photoinitiation using photoinitiators such as lithium phenyl-2,4,6-trimethylbenzoylphosphinate (LAP).^14^ Gelatin methacryloyl (GelMA), a methacrylate-modified gelatin biomaterial, exemplifies this approach and has garnered significant interest in tissue engineering due to its cost-effectiveness, tunable Young’s modulus, biocompatibility, and photo-cross-linkable properties.^15^ GelMA hydrogels loaded with BMSCs have demonstrated potential in regenerating bone defects,^16^ cartilage defects,^17^ dental tissue lesions,^18^ and other combined osteochondral and oro-maxillofacial tissues.^19^

However, limitations regarding LAP post-UV-photoinitiation do exist and have raised concerns regarding the oxidative stresses to which cells encapsulated in such carrier-hydrogels are subjected to. As such, the development of alternatives which might ameliorate the in vitro niche environment when LAP is used is gaining momentum.^20^ Here, we present an alternative platform for constructing synthetic hydrogels for bone tissue engineering based on dendritic–linear–dendritic (DLD) polymers that combine linear PEG with dendritic structures built from 2,2-bis(hydroxymethyl)propionic acid (bis-MPA). The controlled synthesis used to construct the DLDs results in high tunability of the hydrogel’s properties and batch-to-batch consistency. The polyester dendritic family based on bis-MPA has much potential for use in biomedical applications, as it is biocompatible and biodegradable.^21^ Bis-MPA based DLDs have already been used in the creation of antibacterial coatings, where the dendritic systems were decorated with cationic groups and cross-linked through amidation with *n*-hydroxysuccinimide (NHS)-decorated cross-linkers.^22^ The introduction of allyl groups in the outermost generation of the DLDs allows for them to react with thiolated cross-linkers, resulting in hydrogel formation via on-demand photoinduced cross-linking through thiol–ene coupling (TEC) chemistry. TEC is an excellent strategy for hydrogel formation due to its high efficiency, high reaction rate, selectivity, and the possibility to be carried out in water. In addition, the TEC reaction does not negatively impact the biocompatibility of the hydrogel as it does not require metal catalysts and the resulting thioether bond is nontoxic.^23^ The number of allyl groups per DLD is dictated by the generation of their dendritic component, allowing for control over the cross-link density of the hydrogel.

The use of TEC in the presented platform allows for the hydrogel to be injected into the body and then cured in situ, an attractive feature in biomaterial fields.^24^ This ability can be especially interesting for biomaterials in bone regeneration, where the hydrogel would be injected into the bone fracture in order to support and accelerate bone tissue regeneration in shattered fractures above the critical length of natural bone tissue connectivity. Furthermore, from a translation perspective, whenever bone niches are considered, it becomes evident how hydrogels can be used to recapitulate microstructural components. Bone extracellular matrixes consist of mainly a mineral part (70–90%) that comprises hydroxyapatite and an organic part (10–30%) which is mostly composed of collagen (approximately 90%).^25^ As such, both components have attracted extensive research attention as candidates to decorate hydrogels for potential use as bone scaffolds. Indeed, hydroxyapatite has been used to induce osteoconductivity and improve the mechanical properties of hydrogels^26−28^ while other studies have been centered on the formation of hydrogels based on collagen^29,30^ which present good biocompatibility but are limited by the complicated collagen obtention process,^31^ their batch-to-batch consistency, and mechanical properties.^32^ Studies have shown, however, that the combination of both constituents can induce mesenchymal stem cells (MSCs) to differentiate into mature osteoblasts in the absence of inducing factors.^33^

Thus, in this study, we hypothesized that by effective tailoring of hydrogel formulations consisting of allyl functionalized bis-MPA based DLDs and a repertoire of different thiolated cross-linkers, we could maintain viability and activity of BMSCs during in vitro culture after the 3D-encapsulation process. Different formulations have been prepared, namely, to assess the influence of the DLD generation and the hydrogels' dry content regarding parameters such as gel swelling, degradation, and storage modulus. The most promising DLD and dry content were then employed with thiolated cross-linkers of various size and valency to formulate hydrogels for the encapsulation of MSCs, which were evaluated in vitro in terms of cell viability, metabolic activity, and mineralization.

## Experimental Section

### Materials

All materials and solvents used in the synthesis of the DLDs and cross-linkers were purchased from Sigma-Aldrich Sweden AB, TCI Europe NV, and VWR Chemicals and were used as received unless otherwise noted. Bis-MPA was obtained in kind from Perstorp Sweden AB. Hydroxyapatite (HA, microparticle reagent grade powder) and collagen (bovine type 1 powder) were purchased from Sigma-Aldrich Sweden AB. Alpha minimum essential modified Eagle medium (α-MEM) (22571-020), Dulbecco’s phosphate buffered saline (PBS) (14190-144), and fetal bovine serum (FBS) (A52567) were acquired from Gibco. Trypan blue stain (T10282), LIVE/DEAD Cell Imaging Kit (R37601), and PrestoBlue Cell Viability Reagent (A13262) were purchased from Thermo Fischer Scientific USA. Antibiotics (penicillin/streptomycin) were obtained from HyClone. Trypsin/ethylenediaminetetraacetic acid (EDTA) solution (ECM0920D) was purchased from EuroClone. Dexamethasone (D4902), β-glycerophsophate (G9422), l-ascorbic acid (A8960), and Alizarin Red S (A5533) were purchased from Sigma-Aldrich USA. Paraformaldehyde 4% solution (4% PFA) was prepared in house from sodium dihydrogen phosphate monohydrate (Merck; 1.06346.0500), paraformaldehyde (Merck; 1.04005.1000), and disodium hydrogen phosphate dihydrate (Merck; 1.06580.0500).

### Expansion and Culture of BMSCs

Previously isolated and characterized human BMSCs, under ethical approval from the Regional Committee for Medical and Health Research Ethics in Norway (2020/7199/REK sør-øst C), were expanded in culture medium (α-MEM with 10% FBS and 1% antibiotics) in T75 tissue culture flask and incubated at 37 °C in a humidified condition containing 5% CO_2_.^4^ The culture medium was changed three times a week. Cells, at 70–80% confluency, were washed with PBS and detached using Trypsin/EDTA solution for subculture. Cell viability and number were measured using 0.4% Trypan blue stain and a Countess 3 cell counter (Thermo Fischer Scientific). For experiments, BMSCs were used at passages 4 and 5.

### Synthetic Protocols

The synthesis of the DLD polymers, PEG10k-G1-BAPA and PEG10k-G2-BAPA, was carried out following previously described procedures.^34^ Cross-linker PEG2k-SH was prepared as previously published.^35^

### General Procedure for Hydrogel Formation

The formulations of each hydrogel investigated are described in the Supporting Information (Table S1). In general, the DLD and the specific thiolated cross-linker were added to a glass vial in a 1:1 allyl-to-thiol ratio. The mixture was dissolved in deionized (DI) water using a vortex mixer. A solution of the photoinitiator LAP (20 mg/mL in DI water) was then added so that it accounted for 2.4 wt % of the total dry weight of the hydrogel mixture. An initial series of hydrogels was made to investigate the influence of DLD generation and dry content on mechanical properties. This series included either PEG10k-G1-BAPA or PEG10k-G2-BAPA with PEG2k-SH as cross-linker and a dry content of either 10 or 20 wt %. The second series of hydrogels was made to investigate cell viability and consisted of four formulations which included PEG10k-G2-BAPA and either PEG2k-SH (**F1**), dl-dithiothreitol (DTT; **F2**), PEG1k-SH (**F3**), or the tetrathiol cross-linker pentaerythritol tetrakis(3-mercaptopropionate) (PETMP; **F4**) as cross-linker and a dry content of 20 wt %. Curing was achieved with a 20 s duration pulse of high-energy visible (HEV) light from a hand-held light-emitting diode (LED) polymerization lamp (Bluephase 20, Ivoclar Vivadent AG, Leichtenstein), with wavelengths of 385–515 nm (dominant wavelengths of 400 and 470 nm) and an intensity of 1200 mW cm^–2^. These hydrogels were then evaluated with regards to swelling, gel fraction, and rheology. For the hydrogels used for cell viability, metabolic activity, and osteogenic differentiation evaluations, 100% w/v sterile water was used as the solvent, and the cross-linking procedure was conducted under sterile conditions at a temperature of 37 °C. In addition to the DLD containing hydrogels, GelMA 7.5% w/v hydrogel was constructed using an analogous procedure for use as a control, following previously used protocols.^20,36^

### Functionalization of **F2** Hydrogel with Hydroxyapatite (HA) and Collagen

The functionalization of the hydrogel platform with collagen and HA was demonstrated with the **F2** formulation. Collagen or HA was mixed into the **F2** formulation in DI water at different concentrations (collagen or HA wt %) which were defined as the ratio of the mass of collagen or HA to the mass of the remaining components of the formulation, as shown in eq 1. The hydrogel formulation was then cured, as described above.
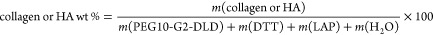
1

### Gel Swelling

Swelling studies were carried out on the hydrogel formulations in PBS at pH 7.4 and 37 °C. The weight of the materials before submersion (*m*_dry_) was obtained after drying the materials in a vacuum oven at 50 °C overnight. The weight of the swollen materials (*m*_swollen_) was obtained at different time points over 28 days and the degree of swelling was calculated using eq 2. Five specimens were evaluated for each hydrogel formulation, except for G1 10 wt %, G2 10 wt %, and **F2** where four specimens were evaluated.
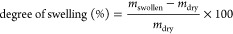
2

### Gel Fraction

Hydrogels were dried in a vacuum oven at 50 °C overnight to obtain *m*_dry_. The hydrogels were submerged in DI water at room temperature with gentle shaking. The water was exchanged after 2 and 4 h. Afterward, the hydrogels were left in DI water overnight. The next day, the hydrogels were leached in 96% ethanol for 4 h, exchanging the ethanol after 2 h. The materials were dried in a vacuum oven at 50 °C overnight and weighted to obtain *m*_leached_. Gel fractions were calculated according to eq 3. The gel fractions of the different formulations can be found in Table S2 in the Supporting Information. Five specimens were evaluated for each hydrogel formulation.
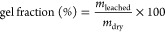
3

### Rheology

Rheological experiments were performed by using a Discovery Hybrid Rheometer II (TA Instruments) with a complete Peltier Plate Temperature System and an 8 mm parallel plate with a geometry gap of 500 μm. Oscillation amplitude sweep measurements were conducted at 37 °C on preswollen hydrogels with a frequency of 1 Hz and oscillation strains ranging from 0.1% to 1000%. Time sweep experiments were performed at room temperature on the formulations using the UV curing accessory from TA Instruments (wavelength range of 320–500 nm, dominant wavelength of 365 nm, intensity 300 mW cm^–2^). Analyses were carried out for 60 s with 15 s of conditioning before the start. Exposure of the hydrogel to UV light began approximately 6 s after the start of the experiment. Five specimens of each hydrogel formulation were evaluated for storage modulus, while three to six specimens were evaluated for UV curing.

### Cell Suspension and Cell Encapsulation

For the hydrogels used for cell viability (**F1**–**F4** and GelMA), suspensions of human BMSCs were incorporated into the hydrogel solutions in α-MEM at a ratio of 1:4 (1 part cell suspension to 4 parts hydrogel precursor solution), following a previously described protocol.^37^ Samples of 50 μL of the cell-loaded hydrogel precursors (containing 5 × 10^4^ cells) were then placed on the upper lid circular rim of a 96-well plate and cured using HEV light, resulting in disc-shaped specimens. The cured specimens were then transferred to 48-well plates. α-MEM was added, and the specimens were incubated under controlled conditions at 37 °C under a 5% CO_2_ atmosphere. The culture medium was refreshed at 48 h intervals. For the evaluation of osteoconductive properties of the hydrogels, samples were divided into two cohorts, placed in a separate 48-well plate, and cultured using normal culture medium or osteogenic medium. Osteogenic medium consisted of α-MEM supplemented with 10 nM dexamethasone, 10 mM β-glycerophosphate, and 173 μM l-ascorbic acid. Both culture media were refreshed every 48 h.

### Assessment of Cell Viability

After cell encapsulation and curing with HEV light, the cell-laden hydrogels (**F1**–**F4** and GelMA) were stained with LIVE/DEAD Cell Imaging Kit after 1, 3, and 7 days. The staining took place over 40 min at room temperature in a dark environment. Cell viability was assessed through image acquisition using an inverted epifluorescent microscope (Eclipse Ti, Nikon, Japan) as a z-stack covering a depth of 500 μm. Image analysis was performed using the “Analyze Particles” function in Fiji/ImageJ, and cell viability quantification was reported as the ratio of living cells to the overall number of cells in the sample. Five specimens were evaluated for each hydrogel formulation.

### Evaluation of Cellular Metabolic Activity

After cell encapsulation and curing with HEV light, the cellular metabolic activity within the hydrogels (**F2**, **F3**, and GelMA) was evaluated at days 1, 3, and 7 using PrestoBlue assay according to instructions from the manufacturer. Ready-to-use Prestoblue solution was added to the medium (10% of the medium volume), and after a 1 h incubation in the dark at 37 °C with 5% CO_2_, reduction of Prestoblue solution was measured by fluorescence (560 nm excitation and 590 nm emission) using a Varioskan LUX multimode microplate reader (Thermo Fisher Scientific). Five specimens were evaluated for each hydrogel formulation.

### Alizarin Red S Staining

BMSCs encapsulated in **F2**, **F3**, and GelMA hydrogels were evaluated with Alizarin Red S staining after days 14 and 21 in osteogenic medium. The hydrogels were fixed in 4% PFA for 40 min, followed by thorough washing in Milli-Q water. Mineral deposition was stained with 0.1% Alizarin Red S stain for 20 min, followed by washing six times with Milli-Q water, with a washing duration of 10 min each.

### Statistics

Data are presented as the mean (standard deviation), and statistical analyses were performed by GraphPad Prism 9 (Dotmatics, USA). For multiple comparisons, the data were evaluated by one-way ANOVA followed by Tukey’s multiple comparisons test. A *p* value of <0.05 was considered statistically significant.

## Results and Discussion

Hydrogels have garnered significant interest in biomedical research due to their impressive capacity to absorb large amounts of water or biological fluids. While traditional hydrogels have faced challenges such as low mechanical strength and suboptimal swelling and degradation profiles, advancements have paved the way for overcoming these limitations.^38^ By modifying the generation of the dendritic–linear–dendritic (DLD) component and the nature of the cross-linker and incorporating various additives, a versatile library of hydrogels with tunable properties has been created. Notably, the cross-linking of these materials was efficiently achieved in water through high energy visible light induced thiol–ene coupling chemistry (HEV-TEC) using lithium phenyl (2,4,6-trimethylbenzoyl) phosphinate (LAP) as a photoinitiator, highlighting the innovative approach and potential of these hydrogels in diverse biomedical applications.

**Figure 1:**
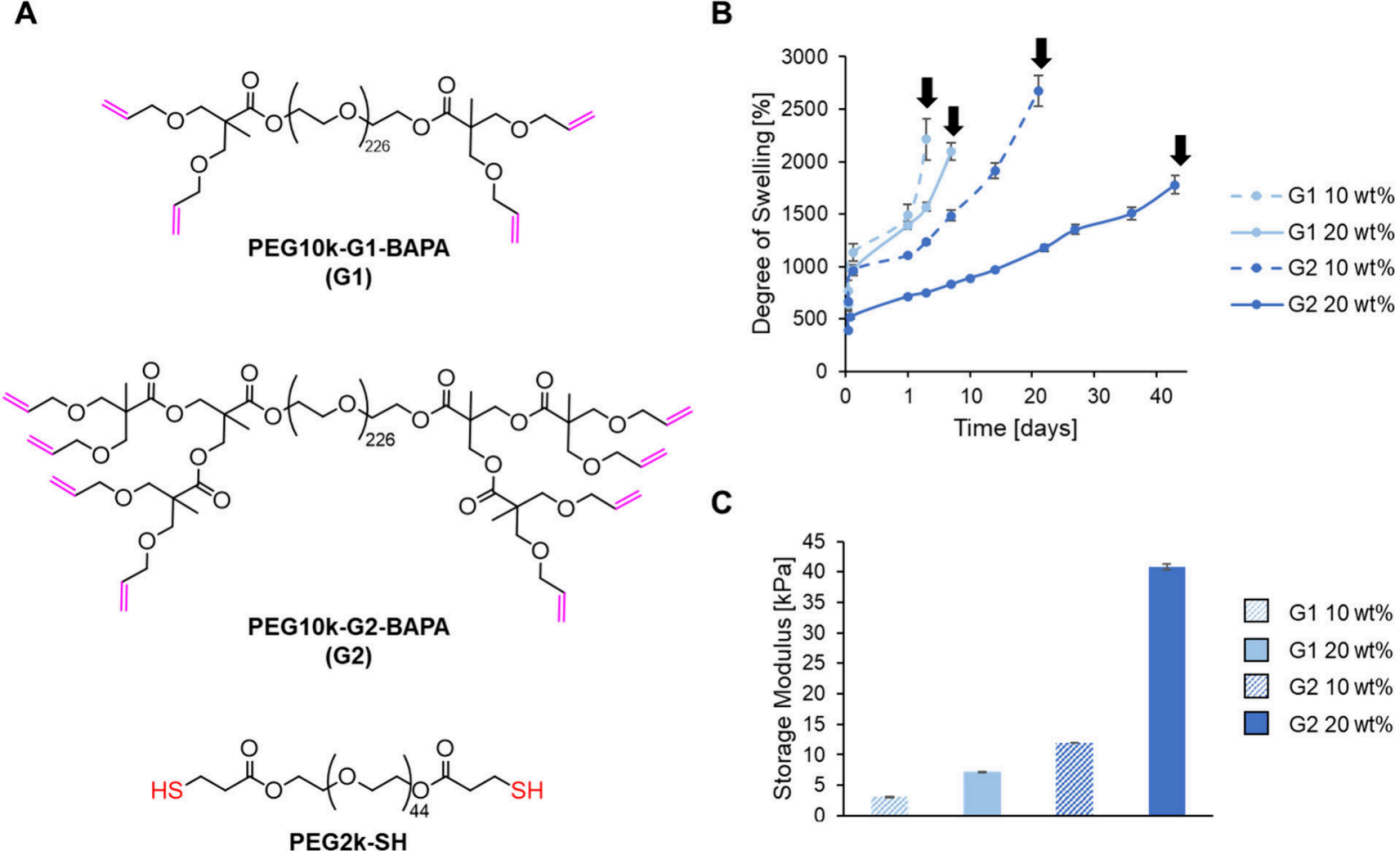
(A) Chemical structures of the DLDs (PEG10k-G1-BAPA, PEG10k-G2-BAPA) and the cross-linker (PEG2k-SH). (B) Degree of swelling in PBS at 37 °C of the hydrogels containing the DLDs and 10 or 20 wt % of dry content. The arrows signal the time points when the gels became too fragile to handle. (C) Storage moduli of the hydrogels in the linear viscoelastic region after swelling overnight in PBS at 37 °C. Values shown are mean values with error bars representing standard deviations (*n* = 5).

The first series of hydrogels was obtained using the first and second generations (PEG10k-G1-BAPA and PEG10k-G2-BAPA) of the DLD polymer and a thiolated, ester-containing PEG of molecular weight 2000 (PEG2k-SH) as a cross-linker (Figure 1A). The influence of the dry content percentage (10 or 20 wt %) on the properties was studied in these formulations. In all cases, the TEC reaction was very efficient. Rheological time sweep experiments showed that a maximum storage modulus was achieved after 6.5 s of UV-light exposure, indicating that the curing process through TEC chemistry was complete (Figure S1). The hydrogel formulations were also efficiently cured within 20 s of exposure from a commercially available hand-held HEV lamp. All the gels had a high gel fraction between 88 (4)%, for the gel made with the G1 DLD (PEG10k-G1-BAPA) at 10 wt % dry content, and 98 (2), for the G1 DLD 20 wt % hydrogel. In general, the rate and degree of swelling were negatively correlated with the cross-link density of the hydrogel, with a faster swelling rate and higher swelling ratio exhibited by the hydrogels made with the G1 DLD than those made with the G2 DLD and with a 10 wt % dry weight content than a 20 wt % dry weight content (Figure 1B). The hydrolytic degradation of the gels was also influenced by the network’s density, with the gels containing the second-generation DLD and the higher dry weight content of 20 wt % remaining stable for longer time periods (Table S3). For example, the G1 10 wt % became impossible to handle after just 3 days, by which time it had swelled to 2213 (198)% of its original weight, while the G2 20 wt % hydrogel was stable for 43 days, by which time it had swelled by 1778 (87)%. The continuous swelling of the hydrogels was likely caused by the hydrolysis of the ester groups within the hydrogel network. The stability of these gels was much higher than previously studied bis-MPA DLD-based hydrogels cross-linked through amidation reactions, which degraded within 24 h.^22,39^ Storage modulus was also correlated with DLD generation and dry weight content, with the PEG10k-G1-BAPA 10 wt % gel being the weakest, with a value of 3.05 (0.06) kPa, and the PEG10k-G2-BAPA 20 wt % gel being the strongest, with a value of 40.9 (0.5) kPa (Figure 1C). These structure–property relationships were similar to those seen in previously studied hydrogels based on bis-MPA DLDs, where increasing the generation of the DLD was also correlated with an increase in storage modulus and decrease in swelling and degradation rate.^39,40^ Previous studies have also shown that the molecular weight of the PEG component of the DLD can be used to tune the cross-linking density and therefore the hydrogel properties, with increasing PEG molecular weight resulting in decreasing storage modulus and increasing swelling and degradation.^40−42^ This approach was not taken in the current study, as the ability to tune the hydrogel properties through design of the cross-linker was focused on instead.

**Figure 2:**
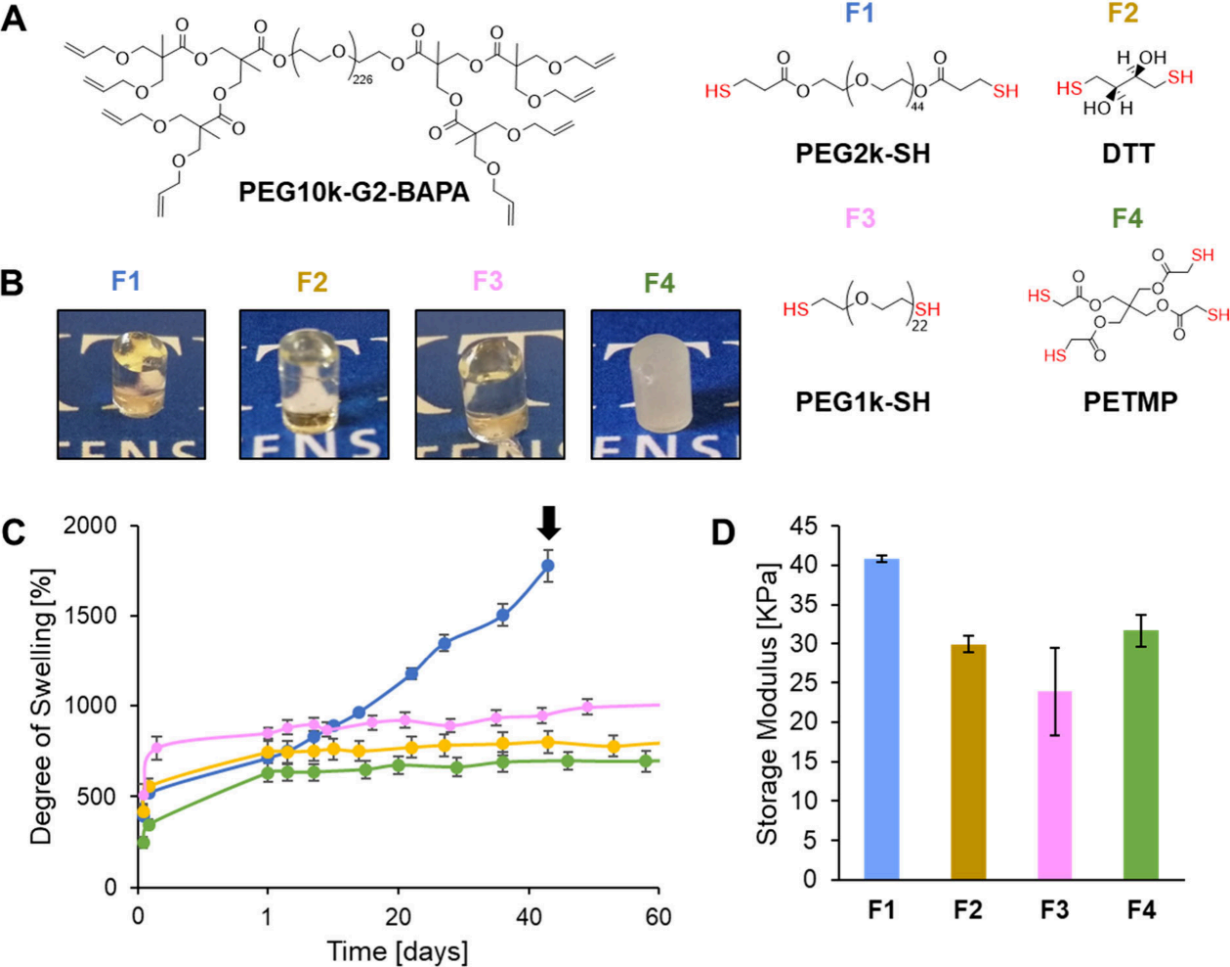
(A) Chemical structures of the PEG10k-G2-BAPA DLD and cross-linkers (PEG2k-SH, DTT, PEG1k-SH, and PETMP) used to make the second series of hydrogels. (B) Images of the cured hydrogel formulations **F1** (PEG2k-SH), **F2** (DTT), **F3** (PEG1k-SH), and **F4** (PETMP). (C) Degree of swelling in PBS at 37 °C of the **F1**–**F4** hydrogels. The **F1** hydrogel became impossible to handle due to degradation after 43 days. (D) Storage modulus in the linear viscoelastic region of the **F1**–**F4** gels after swelling overnight in PBS at 37 °C. Values shown are means with error bars representing standard deviations (*n* = 5).

The conditions that resulted in the most stable hydrogel, PEG10k-G2-BAPA and 20 wt % dry content, were used to investigate how the hydrogel properties could be tuned through the choice of thiolated cross-linker. This hydrogel was chosen for further tuning as it was stable enough to allow for the measurement of osteogenic differentiation of MSCs over a 21-day period, and its modulus was appropriate for use in defects in load-bearing bones^43^ while still being close to the modulus range of 11–30 kPA where MSCs predominately undergo osteogenesis in extracellular matrix.^44^ For comparison with PEG2k-SH, three cross-linkers were considered which differed in terms of valency and susceptibility to hydrolytic degradation: (i) dl-dithithreitol (DTT) and (ii) PEG1k-SH are shorter-chain dithiolated cross-linkers that did not contain hydrolyzable ester groups, and (iii) pentaerythritol tetrakis(3-mercaptopropionate (PETMP) is a tetrathiolated cross-linker (Figure 2A). The hydrogels made with PEG10-G2-BAPA and each of these four cross-linkers at a dry weight content of 20 wt % are referred to as **F1** (PEG2k-SH), **F2** (DTT), **F3** (PEG1k-SH), and **F4** (PETMP). Time sweep experiments showed the **F1**, **F2**, and **F3** hydrogels all cured successfully after 6.5 s of UV light. The storage modulus of the **F4** gel showed a large increase after 6.5 s of UV exposure but continued to steadily increase throughout the remaining 60 s of the experiment (Figure S2). The **F4** gel had a cloudy appearance (Figure 2B), which suggested that PETMP precipitated from the formulation solution and was then entrapped within the hydrogel during curing. NMR analysis of the leach out from the **F4** gel did not detect any PETMP, and the gel fraction of **F4** was 92 (5)%. The **F2** and **F3** hydrogels also had a high gel fraction of 92 (1) and 95 (4)%, respectively (Table S2). The **F2**, **F3**, and **F4** gels were more stable than the **F1** gel, with lower swelling ratios. Unlike the **F1** gel, which continuously swelled until disintegrating at day 43, the **F2**, **F3**, and **F4** gels reached their maximum swelling ratio after 1 day and remained stable for at least 60 days (Figure 2C). For the **F2** and **F3** gels, the higher stability was due to the lack of ester groups in the cross-linker, while for the **F4** gel the lower swelling ratio was due to the higher cross-link density afforded by the tetrathiol cross-linker. The storage modulus in the linear viscoelastic region of the **F2**, **F3**, and **F4** gels showed values of 29.9 (1.1), 23.9 (5.6), and 31.7 (2.0) kPa, respectively, which were lower than the value of 40.9 (0.5) kPa obtained for the **F1** gel (Figure 2D). A comparison the **F1** and **F3** gels suggested a negative correlation between storage modulus and the chain length of the PEG cross-linker, though this relationship was complicated by the higher extent of swelling of the **F3** gel relative to **F1** at day 1, which was when the storage modulus was measured.

### Cell Viability of the **F1**–**F4** Hydrogels

**Figure 3:**
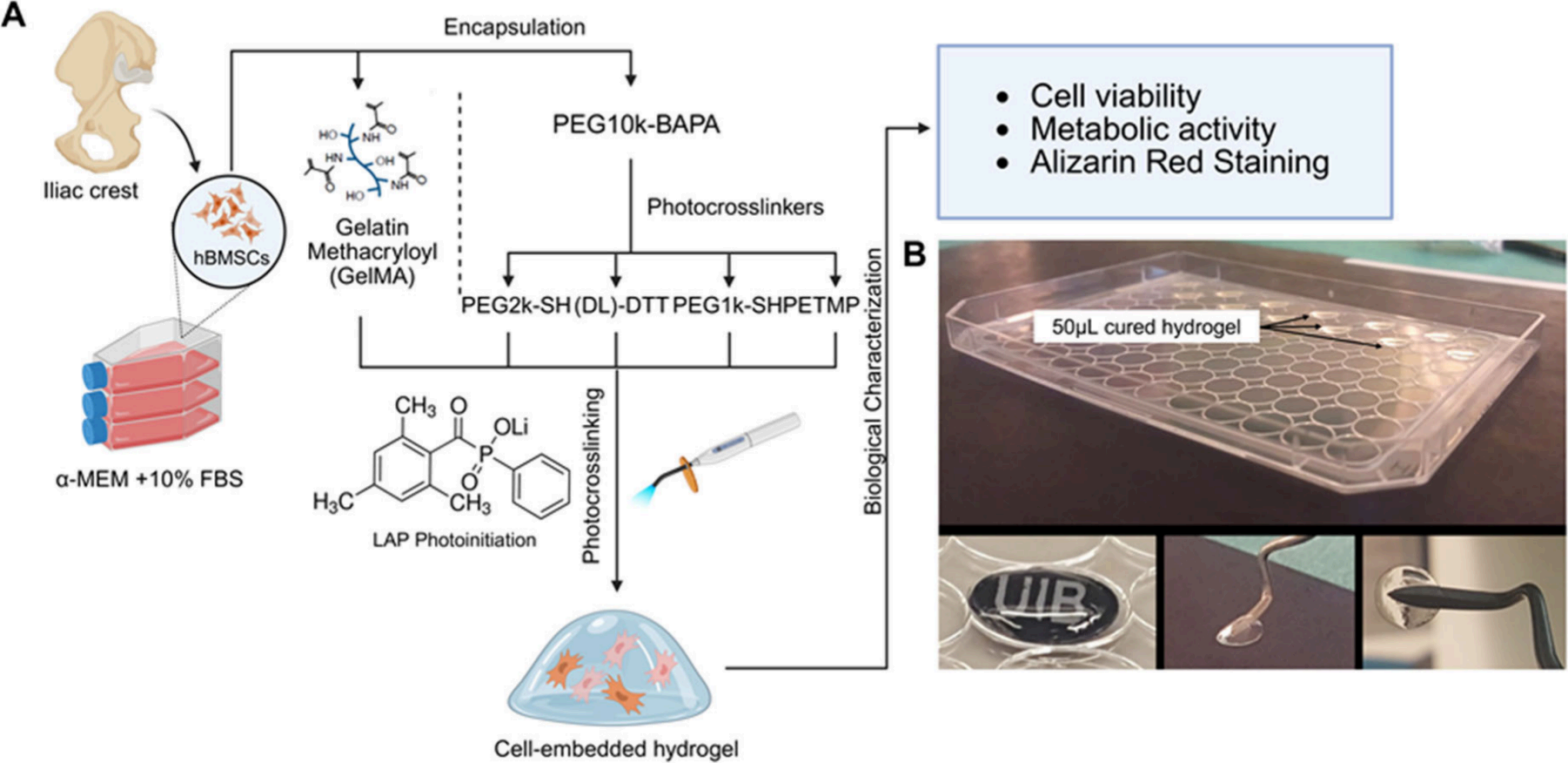
Outline of the biocompatibility and mineralization evaluation of the **F1**–**F4** hydrogels encapsulated with BMSCs. (A) BMSCs were encapsulated within a control group based on GelMA and four experimental groups (**F1**–**F4**) consisting of the same DLD (PEG10k-G2-BAPA) and four different cross-linkers. Samples were molded on a 96-well plate lid and cured with a dental lamp for 20 s and cultured for biocompatibility assays. (B) Depictions of the proof-of concept of the moldability, transparency, and ease of handling of the hydrogels (based on **F1**).

**Figure 4:**
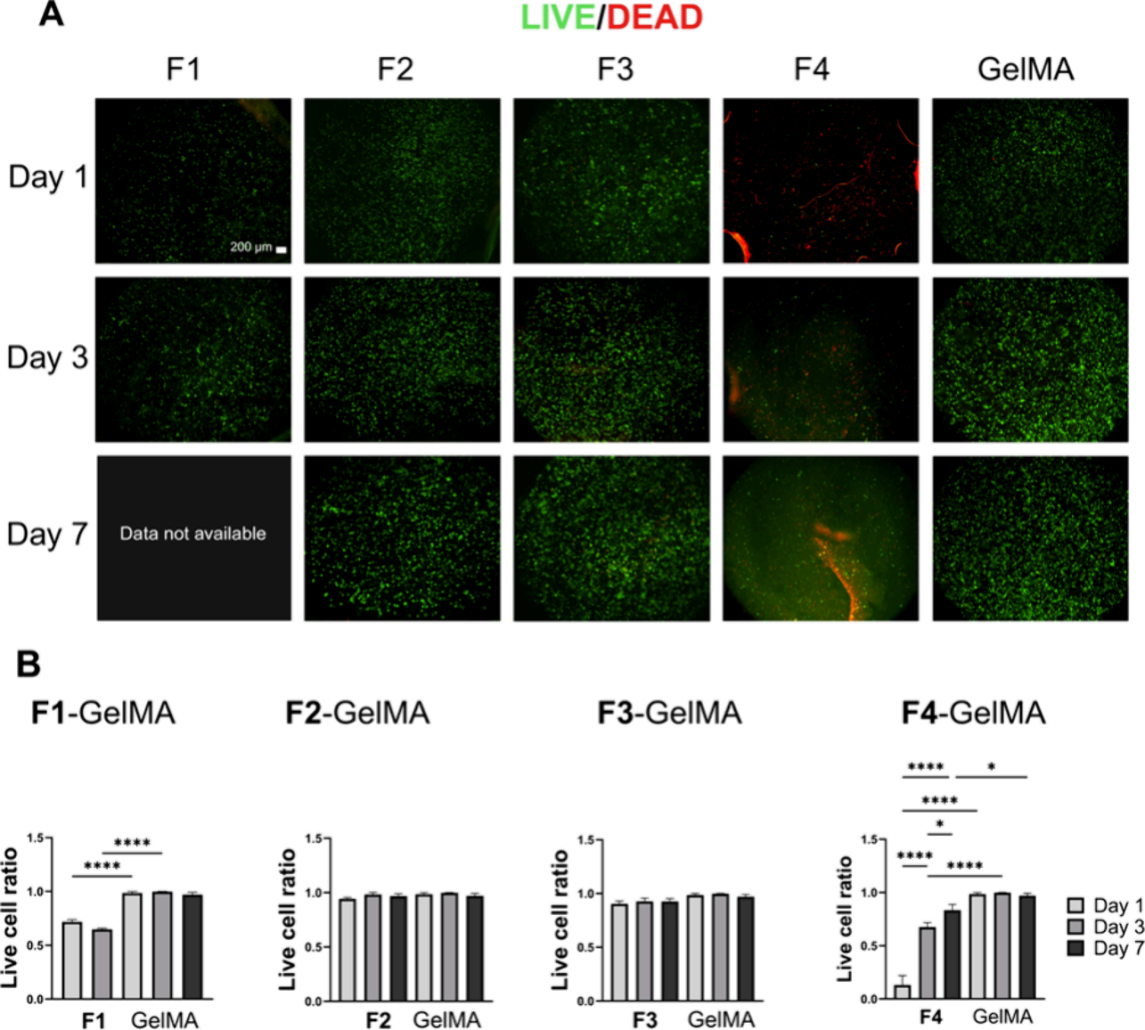
Cell viability by Live/Dead staining, quantification, and comparison between the 4 different experimental hydrogels (**F1**–**F4**) and GelMA. BMSCs encapsulated in both **F2** and **F3** showed remarkable cell viability, denoting the appropriate cytocompatibility properties of these two hydrogels. **F1** data are shown up to day 3 because of excessive swelling and difficult handling. **F4** showed poor cytocompatibility when compared to the other formulations and to GelMA. Imaging acquired via inverted epifluorescent microscope (4×; scale bar 200 μm). Values shown are mean values with error bars representing standard deviations (*n* = 5). *p* values: *p < 0.5%; **p < 0.1%; ***p < 0.01; ****p < 0.001%.

The evaluation of the **F1**–**F4** hydrogels loaded with BMSCs for potential bone regeneration revealed significant differences in their cytocompatibility and stability when compared to GelMA 7.5% w/v. **F1**–**F4** and GelMA were loaded with BMSCs, and the cell viability was tested after 1, 3, and 7 days (Figure 3). After encapsulation and polymerization, cell viability by Live/Dead staining showed that among the four formulations of the developed hydrogel, **F2** and **F3** were comparable to GelMA (Figure 4, Table S4). **F1** showed inferior cell viability compared to **F2**, **F3**, and GelMA. In addition, its substantial swelling and fast degradation made **F1** difficult to handle while changing the medium and processing for staining after 7 days. **F4** demonstrated poor cell viability, with the majority of the cells dead after encapsulation. The poor cell viability of **F4** could have been due to the presence of non-cross-linked and undissolved PETMP, which was suggested by its opaque color (Figure 2B). In addition, it was found that **F4** had a propensity to undergo spontaneous gelation within a short time after the hydrogel had been reconstituted in the water solution. This latter finding was not investigated further. The undissolved PETMP and spontaneous gelation may have resulted in **F4** having an inhomogeneous 3D structure, which could have affected the hydrogel’s permeability to the in vitro culturing environment. This would also have been detrimental to **F4's** cell viability as it would have affected the diffusion of nutrients through the hydrogel. **F2** and **F3** were evaluated further, since these two formulations demonstrated good stability and cytocompatibility.

All of the **F1**–**F4** formulations and GelMA included LAP as a photoinitiator. Overall, LAP can be associated with cytotoxicity, primarily depending on the concentration and cell type-specific sensitivity.^45,46^ The photo-cross-linking process involving LAP induces the release of free radicals during light exposure, which, in turn, can detrimentally affect various biomolecules, including DNA, proteins, and lipids.^47^ Elevated levels of reactive oxygen species (ROS) can induce cellular damage, leading to cell senescence, apoptosis, or necrosis^48,49^ and impair the self-renewal and multidifferentiation capacities which are the important properties of BMSCs for use in tissue engineering applications.^50^ The lower viability observed for **F1** may have been partially due to increased exposure of the BMSCs to harmful radicals as a result of its rapid degradation, while the poor cell viability of **F4** may have also been exacerbated by LAP’s cytotoxic effects. However, the good cell viability findings from day 1 onward of the **F2**, **F3**, and GelMA formulations clearly showed that the BMSCs encapsulated in these hydrogels recovered from any initial damage caused by the activation of LAP; therefore, some initial cytotoxicity from the photoinitiator is permissible depending on the properties of the hydrogel. It should be noted that despite issues with cytotoxicity, LAP does allow for faster polymerization under visible light. While alternative photoinitiators, such as Irgacure 2959 and eosin-Y, are less cytotoxic, they require UV light for curing, which itself poses additional risks for DNA damage and reduced cell viability.^51^ The findings from **F1**–**F4** suggest that while LAP is a convenient and effective photoinitiator for hydrogel cross-linking, its cytotoxic effects must be considered in the design of biomaterials for tissue engineering.^52^ Studies on modulating LAP concentrations or combining it with antioxidants to reduce ROS levels are already showing how to enhance cell viability without compromising polymerization efficiency.^20^ The LAP concentration in the **F1**–**F4** hydrogels was 2.4 wt % of dry weight content, which was chosen based on previous bis-MPA DLD hydrogel formulations.^40^ A reduction in this concentration could increase the polymerization time and risk incomplete curing of the hydrogel. Inadequate cross-linking may reduce cell viability due to the presence of unreacted functional groups and would affect the strength and modulus of the hydrogel. Considering that the hydrogel is intended to be injected into bone defects and cured in situ, these risks may result in poor marginal sealing of the hydrogel in the intended bone restoration. A slower polymerization rate would also be undesirable, given the intended application of the hydrogel. The success of the **F2** and **F3** hydrogels in maintaining stem cell viability highlights the possibility of achieving a balance between the negative and positive effects of the photoinitiator, making them promising candidates for bone regeneration applications.

### Mesenchymal Stem Cell Proliferation in **F2** and **F3**

**Figure 5:**
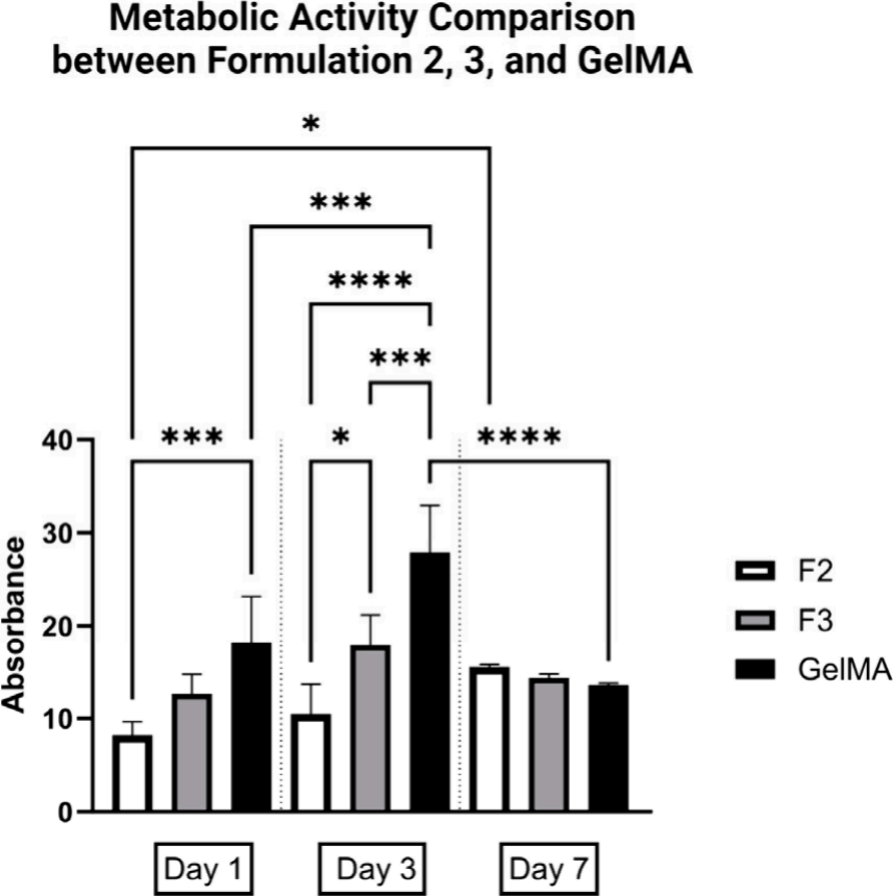
Cell metabolic activity of BMSCs in **F2**, **F3**, and GelMA after 1, 3, and 7 days using PrestoBlue assay. At day 1, GelMA exhibited the highest activity, with **F2** showing lower activity than that of **F3**. By day 3, all formulations, including **F2** and **F3**, demonstrated increased activity compared to day 1, with **F2** and **F3** still showing lower activity than GelMA. By day 7, **F2** showed increased activity relative to **F3** and GelMA, though the differences were not statistically significant. Values shown are mean values with error bars representing standard deviations (*n* = 5). *p* values: *p < 0.5%; **p < 0.1%; ***p < 0.01; ****p < 0.001%.

To study the effects of the photo-cross-linking-induced cellular oxidation and to further validate the cytocompatibility of the **F2** and **F3** gels, cell proliferation was investigated based on cell metabolic activity after 1, 3, and 7 days, with GelMA as a comparison. An increase in cellular metabolic activity was observed from day 1 to day 3 (Figure 5, Table S5), which indicated growth of cells within the hydrogels, confirming the cytocompatibility of these hydrogels. After 7 days, **F2** and **F3** had comparable cellular metabolic activity to GelMA, although this was diminished after 1 and 3 days. Compared with **F2**, **F3** had higher metabolic activity after 1 and 3 days. Overall, the reported findings highlight how the environmental niche properties influenced cell fate. Specifically, the decrease in proliferation and cell metabolic activity observed between days 3 and 7 in **F3** and GelMA can be attributed to the inherent properties of these hydrogels. In accordance with previous reports,^53^ it can be inferred that the inherently higher stiffness of **F3** and GelMA compared to **F2** created a less favorable environment for sustained cell growth and function. This higher stiffness likely impeded the removal of metabolic waste products and hindered the diffusion of nutrients within the hydrogels themselves. As cells continued to grow, the accumulation of waste products and potential nutrient limitations in these stiffer environments led to the observed decrease in cell proliferation and metabolic activity. Furthermore, as already defined, the potential accumulation of ROS within the hydrogels might have caused oxidative stress, further inhibiting cell proliferation and metabolism. In contrast, the adequate stiffness of **F2** likely allowed for better diffusion, supporting continued cell growth and metabolism throughout the experimental period.

### Calcium Deposition from Mesenchymal Stem Cells in **F2** and **F3**

**Figure 6:**
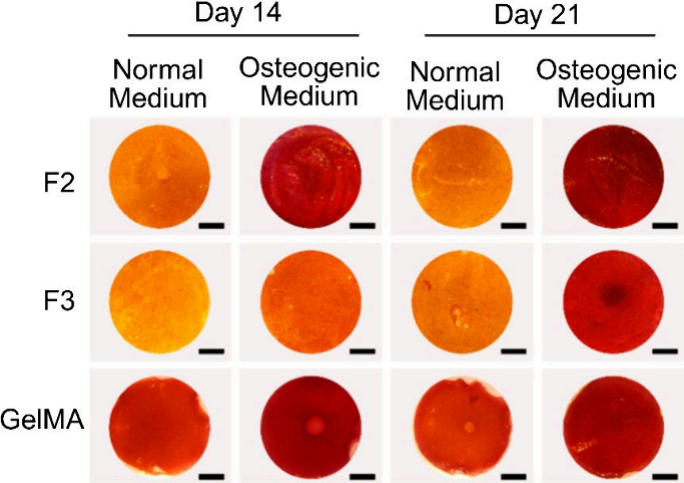
Qualitative assessment of mineral deposition in MSC-encapsulated **F2** and **F3** hydrogels compared to the GelMA control via Alizarin Red staining after 14 and 21 days in normal culture and osteogenic media. At both time points, **F2** demonstrated staining retentions superior to **F3** and comparable to GelMA in both culture conditions, indicating effective mineral deposition (stereomicroscopy, 0.78×, scale bar 2 mm).

The osteoconductive property of **F2** and **F3** and functionality of BMSCs in terms of mineralization induction within the **F2** and **F3** hydrogels were evaluated in comparison to GelMA with Alizarin Red staining after 14 and 21 days (Figure 6). Under osteogenic medium, substantial calcium deposition was detected in **F2**, **F3**, and GelMA, confirming osteogenic differentiation of the encapsulated cells. However, remarkably more calcium deposition was observed in **F2** compared to **F3**, especially after 14 days, indicating that **F2** had better osteoconductive properties than **F3**.

### Functionalization of **F2** with Collagen or Hydroxyapatite (HA)

**Figure 7:**
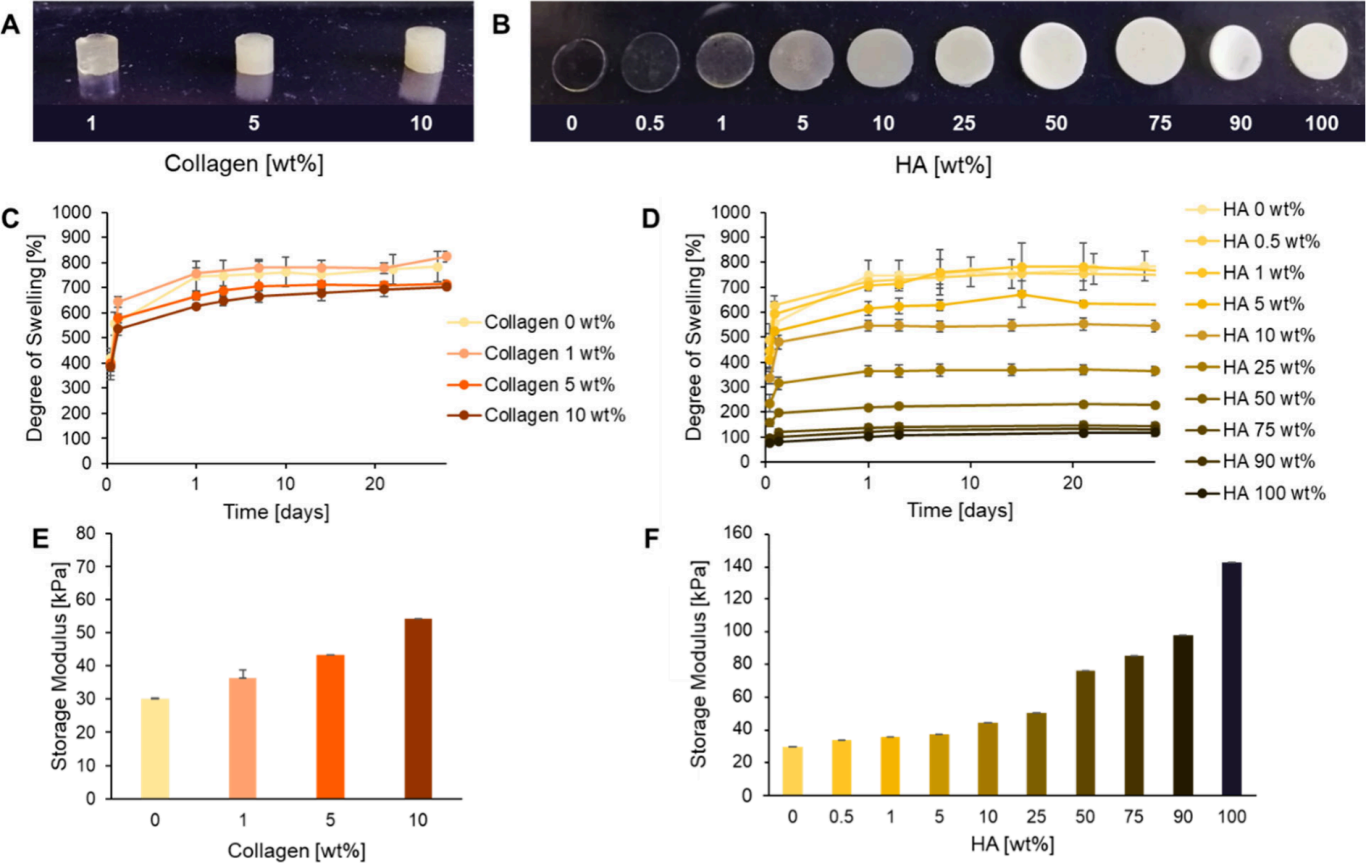
(A, B) Images of the **F2** hydrogel functionalized with 1–10 wt % of collagen and 0–100 wt % of HA. (C, D) Degree of swelling in PBS at 37 °C of the collagen and HA hydrogels. (E, F) Storage modulus in the linear viscoelastic region after swelling overnight in PBS at 37 °C of the collagen and HA hydrogels. Values shown are mean values with error bars representing standard deviations (*n* = 5).

Based on physical and biological evaluations, the **F2** hydrogel was identified as the most promising formulation for use in orthopedic applications. The capacity of the **F2** hydrogel for functionalization with filler particles such as collagen or HA microparticles was determined by adding the fillers to the hydrogel mixture prior to curing. Hydrogels could be successfully cured through TEC chemistry containing collagen content of 1, 5, and 10 wt % or HA content from 0.5 up to 100 wt %, where the mass of HA was equal to the total mass of the remaining components (PEG10k-G2-BAPA, DTT, LAP, and H_2_O). The efficiency of the curing reaction was not impeded by the collagen or HA particles, with rheology showing that all formulations were fully cured within 6.5 s of UV light exposure (Figure S3). Additionally, all gels had a high gel fraction of between 95 (4) and 99 (1)% (Table S2). The opacity of the hydrogels increased with increasing collagen or HA content (Figure 7A-B). For all the collagen and HA containing gels, the swelling ratio plateaued after 1 day in PBS at 37 °C and then remained stable for at least the next 27 days, with no sign of degradation (Figure 7C–D). The magnitude of the swelling ratio was inversely proportional to the collagen or HA content. For the collagen-laden gels, the swelling ratio after 1 day decreased from 746 (60)% for the **F2** gel without collagen, to 757 (23), 667 (15), and 625 (10) % for the 1, 5, and 10 wt % collagen-laden gels, respectively. The swelling ratios of the 1, 5, and 10 wt % HA-laden gels after 1 day were slightly lower, at 706 (17), 614 (27), and 546 (23)%, respectively. The 100 wt % HA gel barely swelled at all, with a ratio of 101 (7)% after 1 day in PBS at 37 °C. The storage moduli of the hydrogels in the linear viscoelastic region were positively correlated with the concentration of collagen or HA, increasing from 29.9 (1.1) kPa for the **F2** gel without any filler to 54.2 (2.6) and 44.4 (0.6) kPa for the 10 wt % collagen or HA gels, respectively (Figure 7E,F). The highest storage modulus, 142.9 (2.8) kPa, was achieved with the 100 wt % HA gel. The high capacity of the **F2** hydrogel for inorganic fillers and biopolymers demonstrated the versatility of the formulation and its potential for formulating complex hydrogels that meet the needs of tissue engineering.

## Conclusions

The combination of the PEG10k-G2-BAPA DLD and various thiolated cross-linkers resulted in formulations that could be cured into hydrogels on demand with HEV light initiated TEC chemistry. The physical properties of the hydrogels could be tuned by the design of the cross-linker, with the cross-linker size, valency, and presence of hydrolyzable ester groups impacting the degree of swelling and storage modulus of the gels. The **F2** hydrogel, containing DTT as a cross-linker, revealed itself to be particularly suitable for the encapsulation of BMSCs, supporting their viability, metabolic activity, and mineralized matrix deposition. The cytocompatibility of **F2** was similar, if not superior, to that of GelMA, which was used as a control. The robust biocompatibility properties of the **F2** hydrogel could significantly enhance the therapeutic efficacy of orthopedic cell therapy. The **F2** hydrogel could also be functionalized with either hydroxyapatite microparticles or collagen, which furthers the potential of using the DLD-based platform to create diverse hydrogels for use in tissue engineering.

## Data Availability

The data sets presented in this study are available publicly in the following open data repository at Zenodo: 10.5281/zenodo.13919543 (PDF, CSV).

## Supporting Information Available

Additional methods and results, including tables showing formulations of all hydrogels evaluated, gel fraction, degree of swelling, storage modulus, cell viability and cell metabolic activity results, and figures showing time sweep rheology data (PDF)

## Abbreviations

α-MEM: alpha minimum essential modified Eagle medium
bis-MPA: 2,2-bis(hydroxymethyl)propionic acid
BMSCs: bone marrow mesenchymal stem cells
DI: deionized
DLD: dendritic–linear–dendritic
DTT: dl-dithiothreitol
EDTA: ethylenediaminetetraacetic acid
FBS: fetal bovine serum
GelMA: gelatin methacryloyl
HA: hydroxyapatite
HEV: high energy visible
LAP: lithium phenyl-2,4,6-trimethylbenzoylphosphinate
LED: light emitting diode
MSCs: mesenchymal stem cells
NHS: *n*-hydroxysuccimide
PBS: phosphate buffered saline
PEG: poly(ethylene glycol)
PETMP: pentaerythritol tetrakis(3-mercaptopropionate
PFA: paraformaldehyde
ROS: reactive oxygen species
TEC: thiol–ene coupling
