# Supplementary material for: Injectable Dendritic Hydrogels Curable by High-Energy Visible Light for Cell Delivery in Bone Regeneration
Source: Chem Mater. 2025 Apr 16;37(9):3284–94. doi: 10.1021/acs.chemmater.5c00063 (PMC12079788; doi:10.1021/acs.chemmater.5c00063)
Supplement: Supplementary file 1 — cm5c00063_si_001.pdf [file cm5c00063_si_001.pdf]

**Supporting Information for**

# Injectable Dendritic Hydrogels Curable by High-Energy Visible Light for Cell Delivery in Bone Regeneration

*Noemi Molina,[1]‡ Francesco Torelli,[2]‡ Samih Mohamed-Ahmed,[2] Daniel J. Hutchinson,[1] Cecilie Gjerde,[2] Ahmad Rashad,[2] Kamal Mustafa,[2] Michael Malkoch[1*]*

*[1] Department of Fibre and Polymer Technology, KTH Royal Institute of Technology, Teknikringen 56-68, 100 44, Stockholm, Sweden.*

*[2] Department of Clinical Dentistry, University of Bergen, Årstadveien 19, 5009, Bergen, Norway*

‡ Authors contributed equally to this work.

[*] Author to whom correspondence should be addressed.

**Table S1.** Formulations used to make 1 mL of each hydrogel using either PEG10k-G1-BAPA (G1), PEG10k-G2-BAPA (G2), the crosslinkers PEG2k-SH, DTT, PEG1k-SH or PETMP, and the fillers collagen or HA at dry contents of 10 or 20 wt%. In all cases, LAP was added as a 20 mg/mL solution in distilled water.

| **Name** | **DLD** | **Crosslinker** | **Dry content** | **m (DLD)** | **m (Cross-linker)** | **m (collagen)** | **m (HA)** | **V (LAP)** | **V ($H_2O$)** |
|---|---|---|---|---|---|---|---|---|---|
| | | | **[wt%]** | **[mg]** | **[mg]** | **[mg]** | **[mg]** | **[µL]** | **[µL]** |
| - | G1 | PEG2k-SH | 10 | 72 | 28 | 0 | 0 | 120 | 780 |
| - | G1 | PEG2k-SH | 20 | 144 | 56 | 0 | 0 | 240 | 560 |
| - | G2 | PEG2k-SH | 10 | 60 | 40 | 0 | 0 | 120 | 780 |
| **F1** | G2 | PEG2k-SH | 20 | 120 | 80 | 0 | 0 | 240 | 560 |
| **F2** | G2 | DTT | 20 | 190 | 10 | 0 | 0 | 240 | 560 |
| **F3** | G2 | PEG1k-SH | 20 | 150 | 50 | 0 | 0 | 240 | 560 |
| **F4** | G2 | PETMP | 20 | 186 | 14 | 0 | 0 | 240 | 560 |
| Collagen 1 wt% | G2 | DTT | 20 | 190 | 10 | 10 | 0 | 240 | 560 |
| Collagen 5 wt% | G2 | DTT | 20 | 190 | 10 | 50 | 0 | 240 | 560 |
| Collagen 10 wt% | G2 | DTT | 20 | 190 | 10 | 100 | 0 | 240 | 560 |
| HA 0.5 wt% | G2 | DTT | 20 | 190 | 10 | 0 | 5 | 240 | 560 |
| HA 1 wt% | G2 | DTT | 20 | 190 | 10 | 0 | 10 | 240 | 560 |
| HA 5 wt% | G2 | DTT | 20 | 190 | 10 | 0 | 50 | 240 | 560 |
| HA 10 wt% | G2 | DTT | 20 | 190 | 10 | 0 | 100 | 240 | 560 |
| HA 25 wt% | G2 | DTT | 20 | 190 | 10 | 0 | 250 | 240 | 560 |
| HA 50 wt% | G2 | DTT | 20 | 190 | 10 | 0 | 500 | 240 | 560 |
| HA 75 wt% | G2 | DTT | 20 | 190 | 10 | 0 | 750 | 240 | 560 |
| HA 90 wt% | G2 | DTT | 20 | 190 | 10 | 0 | 900 | 240 | 560 |
| HA 100 wt% | G2 | DTT | 20 | 190 | 10 | 0 | 1000 | 240 | 560 |

**Table S2.** Physical characterization data of the hydrogels made with either PEG10k-G1-BAPA (G1), PEG10k-G2-BAPA (G2), the crosslinkers PEG2k-SH, DTT, PEG1k-SH or PETMP, and the fillers collagen or HA at dry contents of 10 or 20 wt%: gel fraction, degree of swelling after 1 day in PBS at 37°C and storage moduli in the linear viscoelastic region after swelling overnight in PBS at 37°C. Values are shown as means with standard deviations in parentheses (n =5 for all values except those marked with * where n = 4).

| Name | DLD | Crosslinker | Dry content | Gel Fraction | Degree of Swelling (t = 1 day) | Storage Modulus |
|---|---|---|---|---|---|---|
| | | | [wt%] | [%] | [%] | [kPa] |
| - | G1 | PEG2k-SH | 10 | 88 (4) | 1490 (102)* | 3.05 (0.06) |
| - | G1 | PEG2k-SH | 20 | 98 (2) | 1390 (34)* | 7.17 (0.08) |
| - | G2 | PEG2k-SH | 10 | 94 (2) | 1104 (8) | 12.0 (0.1) |
| **F1** | G2 | PEG2k-SH | 20 | 96 (2) | 714 (10) | 40.8 (0.4) |
| **F2** | G2 | DTT | 20 | 92 (1) | 746 (60)* | 29.9 (1.1) |
| **F3** | G2 | PEG1k-SH | 20 | 95 (4) | 851 (32) | 23.9 (5.5) |
| **F4** | G2 | PETMP | 20 | 92 (5) | 632 (53) | 31.7 (2.0) |
| Collagen 1 wt% | G2 | DTT | 20 | 99 (1) | 757 (23) | 36.3 (0.2) |
| Collagen 5 wt% | G2 | DTT | 20 | 96 (1) | 667 (15) | 43.1 (0.1) |
| Collagen 10 wt% | G2 | DTT | 20 | 96 (2) | 626 (10) | 54.2 (2.5) |
| HA 0.5 wt% | G2 | DTT | 20 | 98 (1) | 722 (24) | 33.7 (0.9) |
| HA 1 wt% | G2 | DTT | 20 | 98 (1) | 706 (16) | 35.6 (0.6) |
| HA 5 wt% | G2 | DTT | 20 | 95 (2) | 614 (27) | 37.3 (1.0) |
| HA 10 wt% | G2 | DTT | 20 | 99 (1) | 546 (23) | 44.4 (0.6) |
| HA 25 wt% | G2 | DTT | 20 | 99 (1) | 364 (22) | 50.5 (0.3) |
| HA 50 wt% | G2 | DTT | 20 | 99 (1) | 218 (10) | 76.0 (0.8) |
| HA 75 wt% | G2 | DTT | 20 | 96 (2) | 137 (14) | 85.1 (0.4) |
| HA 90 wt% | G2 | DTT | 20 | 95 (4) | 120 (7) | 98.1 (0.3) |
| HA 100 wt% | G2 | DTT | 20 | 98 (1) | 101 (7) | 142.9 (2.8) |

**Table S3.** Degree of swelling of the hydrogels made with either PEG10k-G1-BAPA (G1), PEG10k-G2-BAPA (G2) and the crosslinker PEG2k-SH at dry contents of 10 or 20wt% after soaking in PBS at 37°C. Values are given after 1 day and from the final day before the hydrogel became impossible to handle. Values are shown as means with standard deviations in parentheses (n =5 for all values except those marked with * where n =4).

| Name | DLD | Crosslinker | Dry content | Degree of Swelling (t = 1 day) | Degree of Swelling (t = final day) | Final Day Hydrogel could be handled |
|---|---|---|---|---|---|---|
| | | | [wt%] | [%] | [kPa] | [days] |
| - | G1 | PEG2k-SH | 10 | 1490 (102)* | 2214 (198)* | 3 |
| - | G1 | PEG2k-SH | 20 | 1390 (34)* | 2098 (80)* | 7 |
| - | G2 | PEG2k-SH | 10 | 1104 (8) | 2674 (149) | 21 |
| **F1** | G2 | PEG2k-SH | 20 | 714 (10) | 1779 (86) | 43 |

**Table S4.** Cell viability by Live/Dead staining of the hydrogels made with PEG10k-G2-BAPA (G2) and the crosslinkers PEG2k-SH (**F1**), DTT (**F2**), PEG1k-SH (**F3**) or PETMP (**F4**) at a dry content of 20 wt%, and GelMA. No data was collected for **F1** at day 7. Values are shown as means with standard deviations in parentheses (n = 5).

| **Name** | **DLD** | **Crosslinker** | **Dry content** | **Live Cell Ratio** | | |
|---|---|---|---|---|---|---|
| | | | | **Day 1** | **Day 3** | **Day 7** |
| **F1** | G2 | PEG2k-SH | 20.0 | 0.718 (0.021) | 0.648 (0.010) | data not available |
| **F2** | G2 | DTT | 20.0 | 0.943 (0.013) | 0.983 (0.020) | 0.968 (0.021) |
| **F3** | G2 | PEG1k-SH | 20.0 | 0.905 (0.025) | 0.927 (0.030) | 0.926 (0.027) |
| **F4** | G2 | PETMP | 20.0 | 0.131 (0.089) | 0.675 (0.042) | 0.833 (0.056) |
| **GelMA** | - | - | 7.5 | 0.986 (0.014) | 0.998 (0.003) | 0.970 (0.022) |

**Table S5.** Cell metabolic activity of BMSCs in the hydrogels made with PEG10k-G2-BAPA (G2) and the crosslinkers, DTT (**F2**) or PEG1k-SH (**F3**) at a dry content of 20 wt%, and GelMA. Values are shown as means with standard deviations in parentheses (n = 5).

| **Name** | **DLD** | **Crosslinker** | **Dry content** | **Absorbance** | | |
|---|---|---|---|---|---|---|
| | | | | **Day 1** | **Day 3** | **Day 7** |
| **F2** | G2 | DTT | 20.0 | 8.19 (1.45) | 10.44 (3.25) | 15.53 (0.24) |
| **F3** | G2 | PEG1k-SH | 20.0 | 12.62 (2.13) | 17.84 (3.35) | 14.35 (0.43) |
| **GelMA** | - | - | 7.5 | 18.10 (5.09) | 27.91 (5.04) | 13.58 (0.21) |

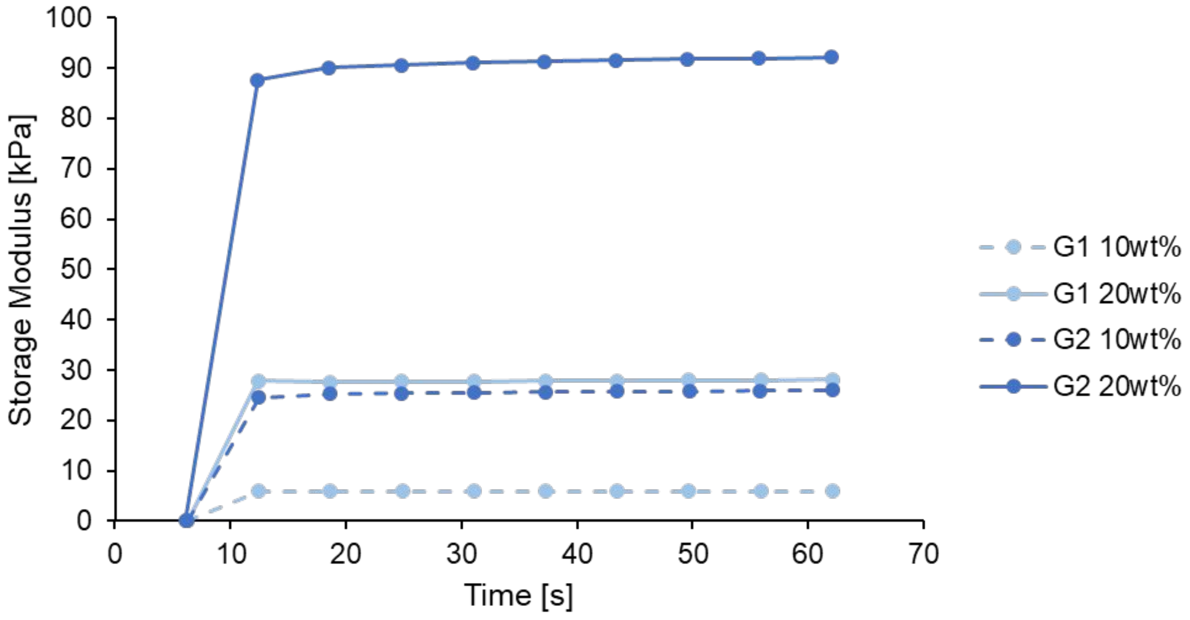


**Figure S1.** Time sweep rheology data of the hydrogel formulations made with either PEG10k-G1-BAPA (G1), PEG10k-G2-BAPA (G2) and the crosslinker PEG2k-SH at dry contents of 10 or 20 wt%, showing the change in storage modulus during curing due to exposure of UV light. The time the UV exposure started (t = 6 s) is indicated by a vertical dotted line. Storage modulus values shown are means (n = 5, except for G1 10 wt% where n = 4).

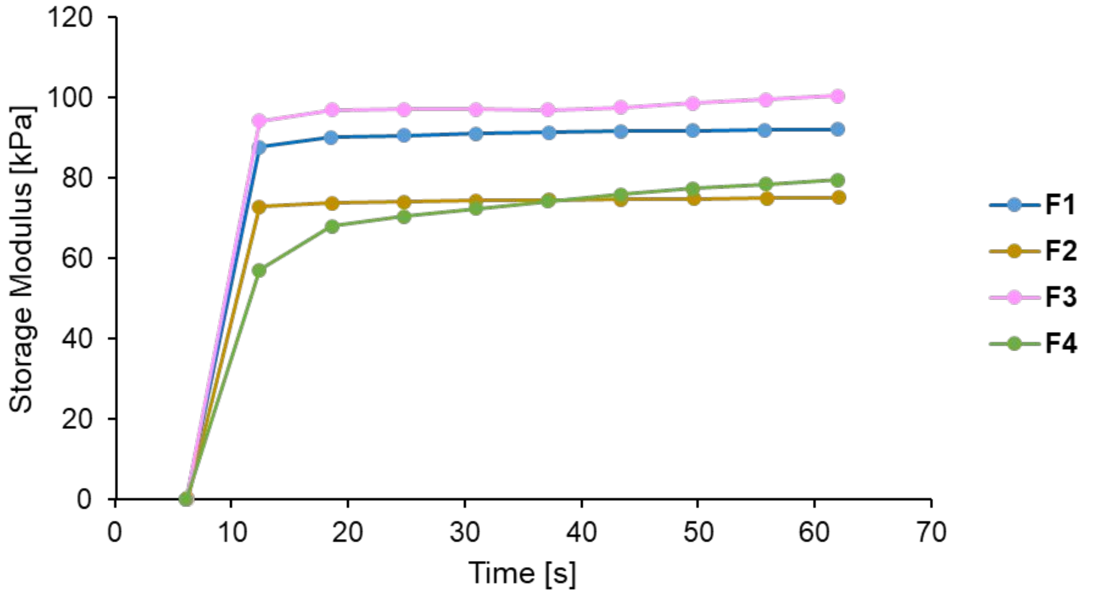


**Figure S2.** Time sweep rheology data of the hydrogel formulations with PEG10k-G2-BAPA (G2) and the crosslinkers PEG2k-SH (**F1**), DTT (**F2**), PEG1k-SH (**F3**) or PETMP (**F4**) at a dry content of 20 wt%, showing the change in storage modulus during curing due to exposure of UV light. The time the UV exposure started (t = 6 s) is indicated by a vertical dotted line. Storage modulus values shown are means (n = 5 for **F1**, n = 4 for **F2**, n =3 for **F3** and n = 6 for **F4**).

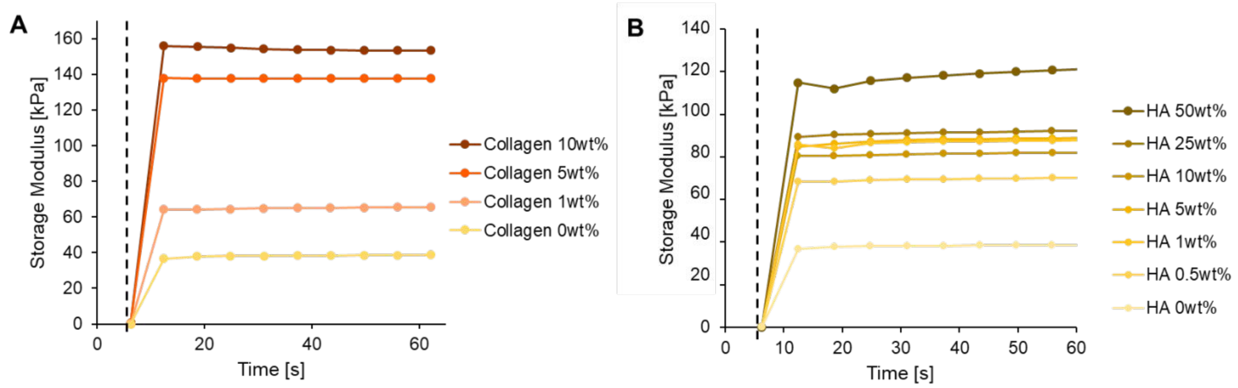


**Figure S3.** Time sweep rheology data of the hydrogel formulations with PEG10k-G2-BAPA (G2) and the crosslinker DTT (**F2**) at a dry content of 20 wt% with (A) collagen or (B) HA, showing the change in storage modulus during curing due to exposure of UV light. The time the UV exposure started (t = 6 s) is indicated by a vertical dotted line. Storage modulus values shown are means (n = 5 for all data).
